# Application of Low-Level Laser Therapy in Endodontics: A Narrative Review

**DOI:** 10.7759/cureus.48010

**Published:** 2023-10-30

**Authors:** Nikhil Mankar, Karuna Burde, Paridhi Agrawal, Manoj Chandak, Anuja Ikhar, Aditya Patel

**Affiliations:** 1 Department of Conservative Dentistry and Endodontics, Sharad Pawar Dental College and Hospital, Datta Meghe Institute of Higher Education and Research (Deemed to be University), Wardha, IND; 2 Department of Public Health Dentistry, Saraswati Dhanwantari Dental College and Hospital, Parbhani, IND

**Keywords:** analgesia, endodontics, dentistry, laser, low-level laser therapy

## Abstract

Low-level laser therapy (LLLT) stands out in the realm of dentistry for its unique attributes that set it apart from traditional therapeutic approaches. This non-invasive and painless modality harnesses the power of low-intensity lasers, offering a distinct advantage in terms of safety and patient comfort. Unlike many conventional methods, LLLT does not rely on pharmaceutical interventions or invasive procedures, making it a gentle yet effective option for various dental applications. Its non-thermal, photobiomodulatory effects on cellular and tissue functions mark a notable departure from the more aggressive treatment modalities commonly associated with dentistry. This article provides an extensive exploration of LLLT's applications in dentistry, focusing on its mechanisms of action and biological effects, and emphasizes the uniqueness of LLLT as a transformative tool in modern dental care.

## Introduction and background

A laser stands as an exceptional and highly specialized source of electromagnetic radiation. The term "laser" is an abbreviation for "Light Amplification by Emission of Radiation" [1]. The utilization of lasers in dentistry commenced after the creation of the ruby laser by Maiman in 1960. In essence, a laser is a device capable of transforming light of various frequencies into colorful radiation, spanning across the visible, infrared, and ultraviolet spectra. Significantly, these radiations possess the unique characteristic of perfect synchronization, allowing them to generate substantial heat and energy when precisely concentrated in close proximity. Within the realm of dental research, significant contributions were made by Stern and Sognnaes [2] and Goldman et al. [3], who were the pioneers in exploring the potential applications of the ruby laser in dentistry. Their initial exploration into laser studies was focused on the hard dental tissues, with the goal of determining the feasibility of utilizing a ruby laser to reduce subsurface demineralization. Remarkably, their efforts produced promising results, as they observed a decrease in enamel permeability to acid demineralization after laser irradiation [4].

The core principle of low-level laser therapy (LLLT) relies on its biostimulatory action. It operates on the premise that exposure to specific wavelengths can induce changes in cellular behavior. Consequently, this leads to enhancements in both cellular metabolism and proliferation. LLLT possesses noteworthy physical attributes, including its ability to combat bacteria, stimulate cell activity, induce tissue ablation, and promote fibroblast growth [5]. Moreover, it enhances hemostasis and the chemotactic activity of leukocytes while also fostering proliferation, differentiation, and calcification of osteoblastic cells. These qualities collectively contribute to its perceived effectiveness in treating endodontic lesions. The inaugural application of lasers in endodontics was documented by Weichman and Johnson in 1971 [6], where they sought to in vitro seal the apical foramen using a high-powered infrared (CO2) laser. While their primary objective remained unattained, their efforts yielded valuable and intriguing data that encouraged further investigation. Consequently, subsequent endeavors focused on apical foramen sealing, employing low-level laser (LLL) technology [7]. The objective of this review is to provide an in-depth exploration of the applications of LLL in the field of endodontics.

Methodology

We conducted searches on both MEDLINE via PubMed and the Cochrane Central Register of Controlled Trials (CENTRAL) database through the Cochrane Library. We utilized various keywords, including "laser," "dentistry," "low level," "applications," "laser therapy," and "endodontics." Additionally, we examined the reference lists of potentially relevant studies to identify any additional research. Our review encompassed studies obtained from these electronic searches and relevant references found within the bibliographies of those studies.

## Review

Classification of laser

Various factors contribute to the classification of lasers, with the primary ones being power, wavelength, and the material used as the laser source. Laser classification is primarily based on power, and they can be categorized into three groups [5]. First, high-power lasers, often referred to as warm lasers or hard lasers, exert effects by heat generation and increasing energy movement within tissues. All these effects encompass evaporation, protein denaturation, necrosis, coagulation, and carbonization. The specific effect achieved depends on the control of the resultant temperature. Typically, high-power lasers have an output exceeding 0.5 watts (W) and find applications in surgical procedures [1]. Second, moderate-power lasers, which include lasers with moderate power levels, achieve therapeutic effects without generating excessive heat. The light of laser has a promoting influence on tissues. The power output of moderate-power lasers falls within the range of 250-500 milliwatts (mW). Third, low-level or cold lasers are the lasers that induce minimal or no thermal effects on tissues. Instead, they stimulate tissues through a process known as photobiostimulation, resulting in gentle and gradual tissue responses [7]. The power output of LLL is typically less than 250 mW. What distinguishes LLL from high-power ones is their ability to trigger photochemical reactions without significant heat generation. It's important to note that the critical factor in distinguishing LLL from high-power one is its power density per unit surface area (e.g., per square centimeter (cm²)) and not their total power. LLL can achieve their stimulating effects with a power density of less than 670 mW/cm² without causing significant heat [5].

Lasers are categorized into four groups based on their wavelengths, which encompass the ultraviolet range (300-400 nm), the visible light range (400-700 nm), the near-infrared (NIR) range (700-1200 nm), and the far-infrared (FIR) range (more than 1200 nm) [1,7]. 

Evolution of LLLT

Early investigations into the utilization of lasers for clinical purposes primarily centered on their use in surgery, particularly with powerful lasers. Nevertheless, in the late 1960s, there was a notable shift toward the exploration of lasers and the use of LLLT in the medical field. During this era, scientists commenced the application of laser irradiation on cells to influence various diseases. Professor Andrew Mester is widely acknowledged as an innovator in the domain of biological stimulation using lasers. His research contributed to the realization that lasers emitting low levels of power could trigger biological effects within their path, exerting non-thermal effects when applied to the skin [8]. In 1968, Mester conducted experiments on cells using argon, helium-neon (He-Ne), and ruby lasers, all operating at low output powers [8]. Additionally, he investigated the histological, immunological, and electron microscopic consequences of laser irradiation on open lesions. The outcomes of these investigation experiments demonstrated improvements in collagen synthesis, the promotion of neovascularization, and increased enzyme synthesis in the areas exposed to laser irradiation.

By the close of 1969, Mester had published an article addressing the effects of LLLT on the enhancement of unhealed or delayed healing wounds. He presented his findings under the title "Application of Low-Level Lasers" [8]. One of the most significant applications of lasers in the field of medicine is LLLT, also called as laser biostimulation, which has garnered considerable recognition from specialists and clinicians in modern times. In this modality involving LLL, the lasers' low output power, wavelength ranging from 630 to 1300 nm, and deeper tissue penetration capability enable them to easily penetrate several centimeters into tissues, reaching cell chromophores like mitochondria, where they exert their effects. Laser therapy operates on the principle that the energy which is absorbed by tissues doesn't generate heat or harm vital tissues. Within this technique, the energy carried by photons of laser is absorbed by cells, resulting in stimulation, a process commonly referred to as photobiostimulation [9].

The remarkable aspect of laser therapy lies in its ability to precisely target specific cells with varying amounts of photons. Despite the wide-range applications of photon-based medicine, some clinicians have yet to grasp the full potential and efficacy of this treatment modality. However, the results of numerous studies unequivocally demonstrate that this treatment method represents a significant advancement in patient care. Photobiotherapy isn't a novel or emerging concept. Sunlight radiation has been employed for therapeutic purposes for many years. However, ordinary light, due to its natural properties and dispersion in all directions, lacks the ability to penetrate living tissues effectively. Therefore, it finds utility primarily in limited dermal treatments for biologic stimulation. Conversely, laser therapy, without the need for drugs, offers the capability to promote wound healing, expedite tissue growth, and stimulate cellular processes [1].

Mechanism of action of LLLT

LLLT has the potential to activate the body's immune system and substantially reduce detrimental inflammatory responses, making it a versatile and valuable tool in the realm of medical treatment. Laser treatment is noted for its ability to sustain the natural process of tissue growth within the body by triggering enzymatic activation. Enzymes are catalysts that expedite chemical reactions, and laser photons exhibit the potential to significantly enhance the efficiency of enzymatic chain processes [10]. In humans, photons get absorbed through the skin, eye, and mucous membranes. Molecules that absorb light can be categorized as photoreceptors and photoacceptors. Photoacceptors, the integral components of crucial cell structures that don't directly respond to light, have a pivotal role in either activating or deactivating reactions once they have been initiated by light. These substances are primarily located within the mitochondrion and the respiratory chain of the cell. A few notable photoacceptors include flavoproteins, riboflavins, porphyrin rings, and cytochrome C oxidase. Photoacceptors exert a governing influence on cellular processes to the cells when they are in a redox state. The mitochondria are the primary site of action for wavelengths in the red and infrared ranges, and these different wavelengths influence the molecules that are accountable for the reduction or oxidation process in the respiratory chain, like cytochromes. Additionally, the cell matrix and membrane are additional sites where infrared and red wavelengths exert their effects. For the ultraviolet range, flavoproteins and riboproteins serve as the photoacceptors [11].

**Figure 1:**
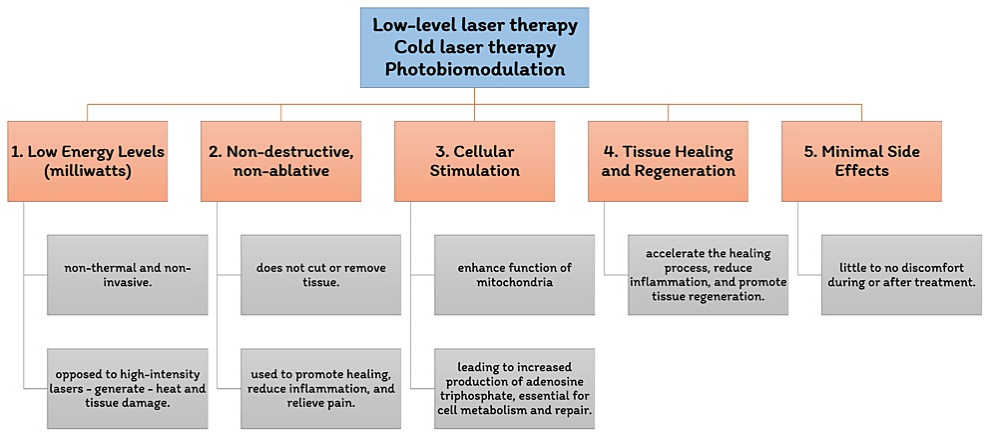
Distinctive features of low-level laser therapy

Cytochrome plays a crucial role in transferring electrons to oxygen. The alteration and movement of electron charges within the mitochondrial membrane create an electrochemical potential that can enhance the potential of the pathway of adenosine triphosphate (ATP) synthesis by up to 300%. This rise in ATP levels subsequently stimulates the processes of RNA and DNA synthesis, ultimately culminating in protein synthesis [12,13]. It's important to note that the impact of laser treatment on cells is not solely determined by the wavelength of laser but by the oxidation state of the chromophores involved. Under normal or near-normal conditions, the cell's response to laser treatment tends to be weaker. However, in a reduced state, the response is stronger, which explains the varied responses observed in cell biology [14]. Furthermore, the irradiation of specific wavelengths can activate photoacceptors at different wavelengths. This infers that alterations in the reduction and oxidation states of the target photoacceptors can influence other spectra's absorption. Cytochrome C oxidase serves as the photoacceptor for the infrared and red spectra, while flavoprotein molecules act as the photoacceptors for the ultraviolet spectrum. Since oxidases only partially absorb light in their activated state, multiple wavelengths can be combined to treat various medical ailments [15]. Figure 1 shows the unique characteristics of LLLT.

Effect of LLLT on cells

The mechanism underlying the impact of LLLT on cells can be divided into two stages: primary reactions and delayed reactions. The first are primary reactions. During laser irradiation, a chain of reactions happens within the cell, resulting in alterations to the cell's redox state, which can involve either oxidation or reduction processes. It's important to note that the redox state significantly influences all metabolic reactions within cells. The cellular response to laser treatment varies depending on the quality of the redox state. When the redox state is normal or near normal, the cellular reaction tends to be less. In contrast, cells that exhibit a propensity for reduction processes tend to respond more favorably to laser treatment. Healthy cells typically exhibit fewer reactions when exposed to laser light, which is a beneficial aspect of laser therapy [5,9].

The second are delayed reactions which refer to responses that persist after the irradiation of the laser. Following the activation of mitochondria, a cascade of biochemical reactions is initiated. This process ultimately results in an increase in the cell's ATP levels. The elevated ATP levels subsequently trigger mRNA activation, DNA replication, and, ultimately, the synthesis of proteins. The effectiveness of laser treatment with different wavelengths can be evaluated by calculating the changes in cell DNA and the amount of synthesized proteins. These synthesized proteins often serve critical functions as neurotransmitters, coenzymes, enzymes, or other substances involved in regulating essential processes. As a consequence of these processes, the effects of laser therapy extend from the cellular level to tissues, organs, and even macrovascular levels [5,9].

Biological consequences of laser treatment on tissue at a physiological level

The tissue's reaction to laser therapy can be categorized into two groups: primary response and secondary response. The primary responses encompass the expansion of blood vessels (vasodilation), enhancement of lymph drainage and circulation of blood, raised activity of neutrophils and fibroblasts, improved cell metabolism, and elevated pain receptor stimulus threshold [15]. The secondary responses include elevated levels of specific prostaglandins like PGL2, known for its anti-inflammatory properties, increased production of immunoglobulins and lymphokines, impacting the immune system, and enhanced production of beta-endorphins and enkephalins, contributing to analgesia. These responses lead to physiological outcomes such as stimulation of the biological system, influence on the anti-inflammatory effect, immune system, and anti-edematous effects, impact on blood vessels and blood circulation, influence on healing of wound, effects on nerves, and analgesic effects [16].

LLLT demonstrates its anti-inflammatory and anti-edema effects throughout all the phases of inflammation. The laser achieves a reduction in swelling, heat, redness, inflammation, and pain through various mechanisms [16] such as enhanced synthesis of PCL2, alteration in prostaglandin synthesis, inhibition of bradykinin synthesis, augmented phagocytosis, vasodilation leading to improved circulation, hiked lymphatic drainage, elevated production of migration inhibitory factor (MIF), and decreased release of histamine.

LLLT also exerts a biologic stimulating effect by modulating the cell's redox system. It shifts anaerobic metabolism toward an aerobic state. Anaerobic metabolism significantly contributes to the creation of pain and inflammation while also slowing down the healing process by generating waste products and reducing pH levels. Therefore, by promoting a shift toward normal and aerobic metabolism, laser therapy induces a beneficial impact [17]. Additionally, infrared and red laser wavelengths stimulate the immune system through various pathways such as activation of T helper and T suppressor cells, immune modulation, enhanced activity of lymphocytes and macrophages, enhanced phagocytosis, elevated levels of immunoglobulins and lymphokines, and strengthening of the complement system [18].

Do LLL have the potential to cause cancer?

One of the extensively investigated domains in laser therapy focuses on wound healing, with groundbreaking studies dating back to as early as 1967, conducted by Mester and Carney [19]. Electromagnetic radiation can pose a potential threat to living organisms if it possesses an intensity high enough to cause burns, typically requiring high wattage, or if it consists of highly energetic photons with short wavelengths. Photons with wavelengths shorter than 320 nm hold the capability to ionize molecules and atoms within tissue, potentially contributing to the development of cancer. The level of risk associated with this depends on both the dosage, measured in joules per cm² of skin, and the power density (intensity) of the radiation [19]. Regarding the potential stimulation of cancer growth in individuals unaware of their condition, there have been no observed detrimental effects when using light with spectrum in the red or infrared range at typical doses applied in laser therapy. However, there is a noticeable distinction between laboratory studies conducted in vitro and real-world experiments involving living organisms, especially with regard to cancer. Studies carried out on rats have shown that small tumors subjected to laser therapy may completely regress, but this treatment appears to have no impact on tumors surpassing a certain size threshold [20].

The electromagnetic spectrum encompasses a wide array of radiation, with wavelengths that vary significantly. Some types of radiation, like gamma rays, have extremely short wavelengths, around 10^-6^ nm, while radio waves can have wavelengths as long as 10^5^ nm. It's important to note that wavelength and frequency are inversely proportional, meaning that shorter wavelengths correspond to higher frequencies [9]. The energy of each photon is directly proportional to its frequency, a relationship described by Planck's constant. Consequently, shorter-wavelength photons carry more energy. High-energy photons can ionize target cells, a process that disrupts chemical bonds in atoms and molecules, potentially leading to mutations. This is why radiations with wavelengths shorter than 320 nm, such as gamma rays, X-rays, and ultraviolet rays, are considered to be carcinogenic. Ultraviolet radiation with wavelengths between 320 and 400 nm can cause skin burns with prolonged exposure but is rarely associated with causing cancer. On the other hand, radiations with longer wavelengths, including radiofrequency, microwave, infrared, and visible light, lack the requisite energy for ionization but can induce excitation and generate heat [11].

Lasers operate within a wavelength range featuring visible light and infrared, typically falling between 600 and 1200 nm. When photons from the visible and infrared range interact with tissues, they can be reflected, transmitted through the tissues, or absorbed. In the process, the power they expend is converted into other forms of energy. As of now, no academic reports have presented evidence of mutagenic effects within this wavelength range [9,21]. Additionally, it's worth noting that the doses used in laser therapy typically range from 0.01 up to a maximum of 50 W/cm², which are often in inhibitory doses. Small changes in DNA have been observed only at much higher doses, around 250 W/cm², which are significantly distant from the therapeutic doses [10]. However, until we achieve absolute certainty through further scientific research and knowledge, it is prudent, especially in cases where the risk of cancer or reproductive organs may be involved, to exercise caution when considering the use of LLL. In the field of laser therapy, a range of lasers has been utilized over the years, including Er:YAG, Nd:YAG, argon, ruby, He-Ne, CO2 lasers, and various types of semi-solid lasers like GaAllnP, GaAlAs, and GaAs. Presently, semi-solid lasers have become the cornerstone of many laser therapy devices. These lasers not only offer favorable biological impact but also exhibit advantages such as being lighter, smaller, cost-effective, and durable (such as Tuner lasers) [9].

Utilization of LLL in dentistry

The application of LLL in dentistry finds a prime niche within the oral cavity due to its ease of access, patient comfort, and specific biological conditions. Within the oral cavity and its vicinity, LLL are deployed for addressing a spectrum of tissues, including soft tissues (such as mucosa, skin, and muscles) and hard tissues (encompassing bones, teeth, and the temporomandibular joint (TMJ)). The presence of such intricate biological structures in this confined space is unparalleled in the human body [7]. Laser therapy is harnessed to treat diverse dental conditions, offering the advantages of minimal anesthesia requirements and time efficiency for patients. It is employed either independently or in conjunction with other treatments like pharmaceuticals, surgery, or physiotherapy. The pivotal role played by lasers lies in their anti-inflammatory, analgesic, and tissue-regenerative biostimulation properties, which facilitate the restoration of tissues to their natural physiological state [10].

In the realm of dentistry, LLL find application across various domains. The choice of treatment method, the selection of an appropriate wavelength, and the determination of effective doses all rest within the purview of the clinician. Moreover, the reaction to various medications and therapies can vary significantly from one person to another. Similarly, the efficacy of laser therapy is contingent upon the condition of the patient's tissues and immune system [13]. Considering the underlying biological mechanisms of laser effects, it is feasible to tailor laser's physical parameters to align with the specific requirements of targeted lesions. Additionally, the frequency of treatment sessions, which can range from daily to every other day, is determined based on the nature of the disease, whether it's acute or chronic, and takes into account the individual's bodily resistance. Given that laser treatment is a relatively young field, the techniques and treatment circumstances are consistently undergoing assessment and enhancement by investigators. Instances of treatment failure primarily stem from the selection of an inappropriate method or insufficient dosage, rather than the inherent inefficacy of laser therapy itself. In a broader context, it is imperative to regularly monitor the treatment regimen and gauge the laser's impact on the patient's condition [21].

Utilizing LLL in endodontic procedures

LLLT for Reducing Dentin Hypersensitivity

Utilizing lasers to alleviate dentin hypersensitivity is a valuable treatment approach. Dentin hypersensitivity manifests as brief, sharp pain arising from exposed dentin when triggered by various stimuli. In the management of dentin hypersensitivity, a variety of anti-sensitivity agents are employed. In more complex cases, such as reversible pulpitis, laser energy can be a beneficial intervention. LLL irradiation focused on the cervical and apical areas of sensitive teeth represents a suitable method for addressing and eliminating sensitivity. The efficacy of this laser treatment primarily hinges on the modifications it induces in pulp nerve transmission [22]. At the cellular level, the laser's impact can be linked to its ability to hinder the transmission of pain signals from peripheral to central regions and the blocking of depolarization in sensory C fibers [23,24].

In a study conducted by Gerschman et al. [25], a LLL with an 830 nm wavelength was employed to alleviate sensitivity in the dentinal area of teeth. Multiple treatment sessions were administered at three intervals. The most substantial reduction in sensitivity occurred following the initial treatment session. Furthermore, there is supporting evidence showing a decrease in dental hypersensitivity immediately upon initiating treatment with He-Ne lasers. This effect continued for up to three months after treatment, with all treated teeth maintaining their vitality. GaAs and GaAlAs lasers possess enhanced penetration capabilities. While the typical focus of irradiation is on the apex region, the use of He-Ne lasers limits irradiation to the anterior cervical region of the teeth. In some instances, it is recommended to apply irradiation to the cervix, pulp, and apex. A tooth that does not respond to 4-6 W of irradiation on its root over two to three sessions is likely in need of root canal therapy [26,27].

LLLT for Managing Periapical Lesions

In a research study, LLLT was employed to expedite the process of wound healing. The results showed a significant reduction in inflammation on days 3 and 7 within the treatment group (p < 0.01). By day 14, the inflammation rate was comparable on both sides (p > 0.05). The utilization of LLLT with optimized parameters has the potential to accelerate the healing of full-thickness wounds [28]. Laser therapy enhances wound healing through a series of mechanisms that come into play during the three phases of inflammation, proliferation, and remodeling. These mechanisms encompass increased synthesis of RNA, DNA, and proteins, promotion of angiogenesis and neovascularization, and acceleration of epithelialization. Additionally, laser therapy exerts anti-inflammatory effects by influencing leukocytes and macrophages, shifting metabolism toward aerobic processes, reducing pain perception, and decreasing the release of pain mediators.

Moreover, it activates the immune system by affecting immunoglobulins IgM-IgG and complements. When LLLT is incorporated into a daily treatment regimen, it stimulates the synthesis of type I collagen fibers, thereby reinforcing scar tissue. The anti-edematous effect of laser energy is rooted in the dilation of lymphatic vessels and reduced permeability of blood vessels. Laser energy also contributes to the regenerative improvement of lymphatic vessels, similar to its effects on veins [9,29]. Metin et al. [30] in 2018 investigated the potential benefits of LLLT in the context of post-endodontic surgery tissue recovery. Their findings indicated that LLLT facilitated the healing of both soft and hard tissues following endodontic surgery. Additionally, it was observed to enhance patient comfort and overall quality of life, especially during the initial stages of the recovery process.

LLLT for Reduction of Pain

Patients may occasionally experience pain on the day following endodontic treatment, particularly in cases of chronic complaints. This discomfort arises as the healing process reactivates and exacerbates the injury [9]. In a research study, LLLT was utilized to alleviate post-endodontic treatment pain, and the results indicated a significant reduction in pain in the LLLT group at four, eight, 12, and 48 hours after the procedure. These findings suggest that LLLT represents an effective non-pharmacological approach to mitigate post-endodontic treatment pain [31]. One of the most widely explored domains in laser therapy is wound healing, an area initially delved into by Mester [21]. In a case study that employed an 810 nm diode laser and LLLT, a 16-month follow-up was conducted to enhance the healing of edema [32].

LLLT as an Analgesic

The analgesic benefits of LLL are among their most noteworthy advantages. These lasers effectively address both acute and chronic pain through a variety of mechanisms. They operate by decreasing the levels of histamine, acetylcholine, serotonin, bradykinin, and prostaglandin E2 while simultaneously enhancing acetylcholine esterase activity. LLL also encourage lymphatic drainage and elevate the levels of ATP and aerobic metabolism. They raise the pain threshold and increase the presence of beta-adrenaline and enkephalins. Furthermore, these lasers help maintain a balance in the activity of adrenaline and noradrenaline while reducing the production of substance P in the spinal posterior horn.

In vivo studies assessing the analgesic effects of LLL on oral nerve fibers have demonstrated a decrease in the frequency of pain signals and an increase in the nerve stimulation threshold. In the realm of pain management using laser therapy, it is possible to irradiate specific acupuncture points, thus obviating the need for needles while achieving similar pain relief results [33].

Potential hazards and adverse effects associated with laser exposure to the body

Laser light poses a potential risk to bodily tissues, especially the eyes and skin, depending on factors like wavelength, power, or output energy. This hazard is not limited to direct contact with tissues but extends to instances where laser light is reflected off reflective surfaces. Additionally, when employing LLL, it's essential to be mindful of potential complications. These may include initial pain sensations due to the transformation of a chronic condition into an acute one, a misleading perception of improvement caused by the laser's analgesic effect before the actual reparative effects become evident, as well as common sensations of fatigue following laser treatment. Furthermore, if laser irradiation occurs in close proximity to blood vessels or if lasers are used intravenously, they can cause vessel dilation, leading to a drop in blood pressure, bouts of vertigo, and temporary darkening of the visual field [34].

Laser therapy comes with a range of contraindications to consider. Firstly, it's not recommended for patients with pacemakers, and if used, caution is necessary. Pregnant women should avoid laser treatment in the uterine region. Laser therapy should be used cautiously in individuals with epilepsy or when the laser frequency falls below 800 Hz. Patients with a history of arrhythmia or chest pain should also steer clear of laser therapy. Glands, such as the thyroid gland, should not be subjected to laser treatment. Tumorous tissues or benign tumors with malignant potential should be treated with caution when it comes to laser therapy. Furthermore, the prescription of laser therapy is not advisable for patients with lupus or those undergoing treatment with light-sensitive substances [35].

On a different note, LLL, often referred to as cold-soft lasers, have been utilized in various countries worldwide for an extended period. These lasers have exhibited a range of beneficial effects, including the reduction of inflammation and pain, acceleration of tissue repair processes, and fortification of the immune system. These advantages have been extensively documented in numerous books and articles. The primary focus of this article is to shed light on some of the advantageous effects of these lasers, particularly in the realm of dental treatments, with a special emphasis on endodontics [36].

## Conclusions

LLL have revolutionized dentistry, particularly in endodontics. These lasers, known for their non-invasive nature and ability to stimulate cellular processes, have shown promise in treating dentin hypersensitivity, expediting wound healing, and reducing post-endodontic pain. However, caution and adherence to safety protocols are crucial due to the potential hazards associated with laser exposure. Despite challenges, ongoing research and clinical trials hold the potential to further enhance the role of LLL in dental care, promising improved treatments and patient outcomes in the future.

## References

[1] Asnaashari M, Safavi N (2013). Application of low level lasers in dentistry (endodontic). J Lasers Med Sci.

[2] Stern RH, Sognnaes RF (1965). Laser effect on dental hard tissues. A preliminary report. J South Calif State Dent Assoc.

[3] Goldman L, Hornby P, Meyer R, Goldman B (1964). Impact of the laser on dental caries. Nature.

[4] Kimura Y, Wilder-Smith P, Matsumoto K (2000). Lasers in endodontics: a review. Int Endod J.

[5] Mokmeli S (2004). The principle of low level laser therapy. Iran, Boshra.

[6] Weichman JA, Johnson FM (1971). Laser use in endodontics. A preliminary investigation. Oral Surg Oral Med Oral Pathol.

[7] Avci P, Gupta A, Sadasivam M, Vecchio D, Pam Z, Pam N, Hamblin MR (2013). Low-level laser (light) therapy (LLLT) in skin: stimulating, healing, restoring. Semin Cutan Med Surg.

[8] Mester E, Mester AF, Mester A (1985). The biomedical effects of laser application. Lasers Surg Med.

[9] Tunér J, Hode L (2010). The new laser therapy handbook : a guide for research scientists, doctors, dentists, veterinarians and other interested parties within the medical field. Sweden, Prima Book AB.

[10] Fekrazad R, Chiniforush N, Bouraima SA, Valipour M, Aslani M, Zare M, Ashtiani Safari O (2012). Low level laser therapy in management of complications after intra oral surgeries. J Lasers Med Sci.

[11] Derr VE, Fine S (1965). Free radical occurrence in some laser-irradiated biologic materials. Fed Proc.

[12] Ben-Dov N, Shefer G, Irintchev A, Wernig A, Oron U, Halevy O (1999). Low-energy laser irradiation affects satellite cell proliferation and differentiation in vitro. Biochim Biophys Acta.

[13] Passarella S, Casamassima E, Molinari S (1984). Increase of proton electrochemical potential and ATP synthesis in rat liver mitochondria irradiated in vitro by helium-neon laser. FEBS Lett.

[14] Bolton P, Young S, Dyson M (1991). Macrophage responsiveness to light therapy with varying power and energy densities. Laser Therapy.

[15] Karu TI (1996). Mechanisms of interaction of monochromatic visible light with cells. Proc SPIE.

[16] Tadakuma T (1993). Possible application of the laser in immunobiology. Keio J Med.

[17] Kovacs IB, Mester E, Gorog P (1974). Laser-induced stimulation of the vascularization of the healing wound. An ear chamber experiment. Experientia.

[18] Mester E (1982). Biostimulating effect of laser beams [Article in German]. Z Exp Chir.

[19] Carney SA, Lawrence JC, Ricketts CR (1967). The effect of light from a ruby laser on the metabolism of skin in tissue culture. Biochim Biophys Acta.

[20] Saldo I (1989). Effects of GaAs-laser on murine sarcoma depends on tomour size. Lasers Surg Med.

[21] Nanami T, Shiba H, Ikeuchi S, Nagai T, Asanami S, Shibata T (1993). Clinical applications and basic studies of laser in dentistry and oral surgery. Keio J Med.

[22] Scherman A, Jacobsen PL (1992). Managing dentin hypersensitivity: what treatment to recommend to patients. J Am Dent Assoc.

[23] Sandford M, Walsh L (1994). Thermal effects during desensitisation of teeth with gallium- aluminium- arsenide lasers. Periodontol.

[24] Featherstone JD, Nelson DG (1987). Laser effects on dental hard tissues. Adv Dent Res.

[25] Gerschman JA, Ruben J, Gebart-Eaglemont J (1994). Low level laser therapy for dentinal tooth hypersensitivity. Aust Dent J.

[26] Tsuchiya K, Kawatani M, Takeshige C, Matsumoto I (1994). Laser irradiation abates neuronal responses to nociceptive stimulation of rat-paw skin. Brain Res Bull.

[27] Matsui S, Tsujimoto Y, Matsushima K (2007). Stimulatory effects of hydroxyl radical generation by Ga-Al-As laser irradiation on mineralization ability of human dental pulp cells. Biol Pharm Bull.

[28] Alipanah Y, Asnaashari M, Anbari F (2011). The effect of low level laser (GaAlAs) therapy on the post-surgical healing of full thickness wounds in rabbits. Med Laser Application.

[29] Ando T, Noguchi I, Satoh Y (1985). Use of soft lasers in dentistry [Article in Japanese]. Shikai Tenbo.

[30] Maia ML, Bonjardim LR, Quintans Jde S, Ribeiro MA, Maia LG, Conti PC (2012). Effect of low-level laser therapy on pain levels in patients with temporomandibular disorders: a systematic review. J Appl Oral Sci.

[31] Asnaashari M, Mohebi S, Paymanpour P (2011). Pain reduction using low level laser irradiation in single-visit endodontic treatment. J Lasers Med Sci.

[32] Asnaashari M, Asnaashari N (2011). Clinical application of 810nm diode laser and low level laser therapy for treating an endodontic problem a case presentation. J Lasers Med Sci.

[33] Schlager A, Offer T, Baldissera I (1998). Laser stimulation of acupuncture point P6 reduces postoperative vomiting in children undergoing strabismus surgery. Br J Anaesth.

[34] Metin R, Tatli U, Evlice B (2018). Effects of low-level laser therapy on soft and hard tissue healing after endodontic surgery. Lasers Med Sci.

[35] Gündoğar H, Şenyurt SZ, Erciyas K, Yalım M, Üstün K (2016). The effect of low-level laser therapy on non-surgical periodontal treatment: a randomized controlled, single-blind, split-mouth clinical trial. Lasers Med Sci.

[36] Domah F, Shah R, Nurmatov UB, Tagiyeva N (2021). The use of low-level laser therapy to reduce postoperative morbidity after third molar surgery: a systematic review and meta-analysis. J Oral Maxillofac Surg.

